# Exploration of the Supercapacitive Performance of 3D Flower-like Architecture of Quaternary CuNiCoZnO Developed on Versatile Substrates

**DOI:** 10.3390/mi16060645

**Published:** 2025-05-28

**Authors:** Priya G. Gaikwad, Nidhi Tiwari, Rajanish K. Kamat, Sadaf Jamal Gilani, Sagar M. Mane, Jaewoong Lee, Shriniwas B. Kulkarni

**Affiliations:** 1 Material Research Laboratory, Department of Physics, The Institute of Science, Dr. Homi Bhabha State University, Mumbai 400032, Maharashtra, India; gaikwadpriya347@gmail.com (P.G.G.);; 2 Faculty of Physics, Thakur College of Engineering and Technology, Kandivali (E), Mumbai 400101, Maharashtra, India; 3 Department of Pharmaceutical Sciences, College of Pharmacy, Princess Nourah bint Abdulrahman University, P.O. Box 84428, Riyadh 11671, Saudi Arabia; 4 Department of Fiber System Engineering, Yeungnam University, Gyeongsan 38541, Gyeongbuk, Republic of Korea

**Keywords:** facile synthesis, quaternary metal oxide, 3D architecture, different substrates, charge storage mechanism

## Abstract

The demand for high-performance supercapacitors has driven extensive research into novel electrode materials with superior electrochemical properties. This study explores the supercapacitive behavior of quaternary CuNiCoZnO (CNCZO) films engineered into a three-dimensional (3D) flower-like morphology and developed on versatile substrates, including carbon cloth, stainless steel mesh, and nickel foam. The unique structural design, comprising interconnected nanosheets, enhances the electroactive surface area, facilitates ion diffusion, and improves charge storage capability. The synergistic effect of the multi-metallic composition contributes to remarkable electrochemical characteristics, including high specific capacitance, excellent rate capability, and outstanding cycling stability. Furthermore, the influence of different substrates on the electrochemical performance is systematically investigated to optimize material–substrate interactions. Electrochemical evaluations reveal outstanding specific capacitance values of 2318.5 F/g, 1993.7 F/g, and 2741.3 F/g at 2 mA/cm^2^ for CNCZO electrodes on stainless steel mesh, carbon cloth, and nickel foam, respectively, with capacitance retention of 77.3%, 95.7%, and 86.1% over 5000 cycles. Furthermore, a symmetric device of CNCZO@Ni exhibits a peak specific capacitance of 67.7 F/g at a current density of 4 mA/cm^2^, a power density of 717.4 W/kg, and an energy density of 25.6 Wh/kg, maintaining 84.5% stability over 5000 cycles. The straightforward synthesis of CNCZO on multiple substrates presents a promising route for the development of flexible, high-performance energy storage devices.

## 1. Introduction

The global energy crisis has escalated, marked by soaring energy costs and growing anxiety over the ability to meet the power demands of households, industries, and entire nations. This crisis is driven by two primary factors. The first is demographic and economic: as the global population continues to grow, so too does individual demand for goods and services, which in turn increases overall energy consumption. This demand surge would be less problematic if energy resources were both ample and clean. However, the environmental toll of greater energy use complicates the issue. The second factor involves the ecological consequences of fuel extraction, distribution, and consumption, processes that have significantly degraded environmental conditions [1,2,3]. Given these dynamics, there is a pressing need to create energy systems that are not only highly efficient but also require minimal maintenance. Addressing this demand has become a critical focus for researchers worldwide, who are working intensively to develop next-generation energy technologies.

Supercapacitors (SCs) are emerging as advanced electrochemical energy storage (EES) systems capable of addressing upcoming energy challenges and contributing to environmental sustainability. Their exceptional power density and distinct charge storage processes have attracted considerable attention, distinguishing them from traditional battery technologies. Based on their underlying energy storage mechanisms, supercapacitors are typically divided into two main types: electric double-layer capacitors (EDLCs) and pseudocapacitors. A combination of these two results in hybrid capacitors, which aim to harness the advantages of both [4,5,6,7]. Carbon-based materials are widely employed in EDLCs due to their excellent cycling stability and favorable charge-discharge efficiency, though they generally offer limited energy density. In contrast, pseudocapacitors frequently utilize transition metal oxides, which deliver higher energy storage capacity owing to their Faradaic charge storage behavior. Despite their relatively shorter lifespan in cyclic operations, these materials exhibit significantly greater capacitance [8,9]. Numerous studies have reported the use of metal oxides, hydroxides, and intrinsically conductive polymers as electrode materials for pseudocapacitors [10,11,12]. Nanostructured forms of metal oxides are especially beneficial, as they provide larger surface areas and facilitate more efficient ion and electron transport, thereby boosting the specific capacity of the devices [13,14]. These materials also demonstrate pronounced redox reactivity and are capable of achieving high theoretical capacitance values. Among them, nickel and cobalt-based compounds are particularly favored for their superior performance in supercapacitor applications.

Multi-metal oxides comprising binary, ternary, and quaternary compositions have attracted attention due to their ability to support multiple redox reactions. This advantage arises from the coexistence of several redox-active metal species, each capable of undergoing valence changes. Compared to single-metal oxides, multi-metal oxides often demonstrate enhanced redox properties, making them promising candidates for electrode materials. For instance, Jadhav et al. [15] reported the hydrothermal synthesis of a hierarchical nickel oxide structure combining one-dimensional and three-dimensional features with doping with different metals. This material exhibited a specific capacitance of 639.3 F/g at a scan rate of 5 mV/s, with a corresponding specific energy of 74.13 Wh/kg and a specific power of 2.07 kW/kg. It is noted that NiCo_2_O_4_ electrodes surpass pure NiO in electrochemical performance. The NiCo_2_O_4_ electrodes displayed higher specific capacitance, improved pseudocapacitance characteristics, better rate performance, and enhanced cycle stability, attributed to their multi-valent cationic composition and potential for structural tuning [16]. Zhang et al. successfully fabricated nanoneedle arrays of NiCo_2_O_4_ directly on carbon cloth substrates. These structures demonstrated an impressive specific capacitance of 932 F/g in a 2 M KOH electrolyte at a current density of 2 A/g, underscoring their suitability for high-performance energy storage devices [17]. Tiwari et al. [18] synthesized ZnCo_2_O_4_ electrode material using a hydrothermal method, directly depositing it onto a stainless-steel substrate. The resulting electrode exhibited a specific capacitance of 593 F/g at a scan rate of 10 mV/s. Additionally, the zinc cobaltite electrode demonstrated a high Coulombic efficiency of 96.50%, indicating excellent charge–discharge reversibility.

In pursuit of enhanced electrode capabilities, significant attention has been directed toward the development of ternary and quaternary metal oxides composed of three or more distinct metallic elements. These complex oxides offer numerous benefits, including the presence of multiple redox-active centers, elevated pseudocapacitive responses, extended cycling stability, and increased flexibility for both structural and compositional tuning [19,20]. For example, Duan et al. [21] reported the successful fabrication of ternary Ni–Co–Mo oxyhydroxide nanoflakes directly grown on carbon cloth, marking a significant advancement in high-performance supercapacitor electrode design. These nanostructures exhibited impressive specific capacitance and excellent cycling durability, underlining their suitability for energy storage applications. In another innovative approach, Acharya et al. [22] developed Zn–Ni–Co ternary oxide nanowire arrays on nickel foam using a two-step hydrothermal synthesis process combined with theoretical modeling. This method led to electrodes with improved electrochemical characteristics, reflecting the potential of ternary oxides in next-generation supercapacitors. A notable study by Kiey et al. [23] involved the synthesis of nanocrystalline transition metal ferrites, namely CoFe_2_O_4_, CuFe_2_O_4_, and a composite form Co/CuFe_2_O_4_, via a citrate precursor method. These materials were employed as electrode candidates for supercapacitor systems. Among their electrochemical attributes, the nanoparticles delivered a peak-specific capacitance of 893 F/g at a scan rate of 5 mV/s. Furthermore, the Co–Cu ferrite electrode demonstrated excellent durability, retaining 90% of its capacitance after 3000 charge-discharge cycles at a current density of 1 A/g. Zhang et al. [24] synthesized a three-dimensional, porous quaternary oxide consisting of zinc, nickel, aluminum, and cobalt (ZNACO), forming nanosheets directly on a nickel foam substrate. This architecture achieved a notable specific capacity of 839.2 C/g at 1 A/g and retained 82% of its capacity, indicating strong structural and electrochemical stability. Similarly, Halder et al. [25] introduced a cost-efficient hydrothermal synthesis route to produce a 3D quaternary metal oxide composed of copper, nickel, cerium, and cobalt (Cu–Ni–Ce–Co). This material delivered an ultrahigh specific capacitance of 2696 F/g at a current density of 1 A/g and maintained 86.5% of its capacitance after 3000 charge-discharge cycles, showcasing both high performance and long-term reliability. Further pushing the frontier, Mohanty et al. [26] employed an induction melting technique to produce a high-entropy alloy (HEA) incorporating Fe, Co, Ni, Cu, and Zn. The resulting material was applied as a positive electrode in an asymmetric supercapacitor, achieving a peak gravimetric capacitance of 325.17 F/g at 1 A/g. Additionally, the device demonstrated an energy density of 23.82 Wh/kg at a power density of 325 W/kg, indicating its promise for practical energy storage systems.

The collective findings from prior studies indicate that, in addition to the incorporation of multiple metal components, the construction of a well-organized hierarchical structure is equally vital in influencing the electrochemical behavior of electrode materials. Such architectures enhance ion diffusion, facilitate efficient electron transport, and increase the exposure of electrochemically active sites. The formation of hierarchical architectures is strongly influenced by several key parameters, including the choice of synthesis method, reaction temperature, solution pH, reaction duration, composition, and other processing conditions [27,28]. Therefore, it is essential to investigate the energy storage potential of multi-metal oxide materials by taking into account multiple influencing factors, such as composition, structural morphology, and synthesis conditions. Another important factor in practical supercapacitor design is the compatibility of active materials with various substrates. The ability to directly grow or integrate electrode materials onto versatile substrates, such as metal foams, conductive fabrics, and flexible polymers, opens up new possibilities for wearable and flexible energy storage devices. The substrate not only supports the mechanical integrity of the electrode but also influences the nucleation, growth, and adhesion of the active material, ultimately affecting its electrochemical performance.

In this study, we employed a straightforward hydrothermal synthesis approach to fabricate Co-Ni-Cu-Zn oxide nanostructures directly on carbon cloth, stainless steel mesh, and nickel foam substrates, eliminating the need for any binder materials. Each metal is supposed to contribute with distinct functionalities: Cu enhances electrical conductivity; Ni and Co provide abundant redox-active sites; and Zn contributes to structural stability and corrosion resistance. Furthermore, carbon cloth, stainless steel (SS) mesh, and nickel (Ni) foam serve as versatile substrates for supercapacitor electrodes, each offering unique benefits. These include flexibility and lightweight characteristics (carbon cloth), cost-effectiveness and mechanical stability (SS mesh), and high surface area with excellent electrical conductivity (Ni foam), thereby addressing diverse performance and application-specific requirements. By systematically investigating the structural, morphological, and electrochemical characteristics of this quaternary material, we aim to elucidate the interrelationship between material nanostructure, substrate effects, and capacitive performance. The findings of this work contribute to the growing body of knowledge on multi-metal electrode materials and offer valuable insights into the rational design of advanced supercapacitors for future energy storage applications.

## 2. Experiment

### 2.1. Materials

All analytical chemicals, including cobaltous nitrate hexahydrate Co(NO_3_)_2_·6H_2_O, Nickel nitrate hexahydrate Ni(NO_3_)_2_·6H_2_O, cupric nitrate trihydrate Cu(No_3_)_2_·3H_2_O, zinc nitrate hexahydrate Zn(No_3_)_2_·6H_2_O, and urea NH_2_CoNH_2_, were purchased from S D Fine-chem Limited, Mumbai, India, and no additional purification was necessary. Current collectors used for active material deposition included carbon cloth (CC), stainless steel (SS) mesh, and nickel foam (Ni), each with dimensions of 1 cm × 4 cm. The carbon cloth was procured from Vinpro Technologies, Hyderabad, India, the stainless steel mesh was sourced from a Zain Corporation, Pune, India, and the nickel foam was obtained from MTI Corporation, Seoul, Republic of Korea. The cleaning procedure for the substrates involved ultrasonic treatment in a solution comprising ethanol and deionized water. In the case of nickel foam, an additional cleaning step was performed using diluted hydrochloric acid following its initial ultrasonication in distilled water to ensure the complete removal of surface impurities. After cleaning, all substrates were dried at 60 °C for 24 h. Double-distilled water was used as the solvent throughout the synthesis process.

### 2.2. Synthesis of CNCZO on Different Substrates

The quaternary CNCZO was deposited on different precleaned substrates (CC, SS mesh, and Ni-foam) via a hydrothermal approach. Initially, 2 mM each of cobalt nitrate hexahydrate, nickel nitrate hexahydrate, cuprous nitrate trihydrate, and zinc nitrate hexahydrate, along with 10 mM of urea, were dissolved in 50 mL of deionized water. This solution was stirred for 30 min using a magnetic stirrer to ensure homogeneity. The pH was adjusted to neutral (pH 7) by gradually adding ammonium hydroxide. Once the solution was ready, CC, SS-mesh, and Ni-foam substrates were immersed in the precursor solution and sealed in a Teflon-lined autoclave. Hydrothermal treatment was conducted at 150 °C for four hours. After the reaction period, the system was allowed to cool naturally to ambient temperature. The resulting CNCZO-coated substrates were then thoroughly rinsed with deionized water to remove any unreacted species and subsequently dried at 60 °C. The dried electrodes were further calcined at 250 °C for two hours to enhance crystallinity and adhesion. The final mass loading of the active CNCZO material was determined to be 1.0 mg/cm^2^ on SS Mesh, 1.6 mg/cm^2^ on carbon cloth, and 2.3 mg/cm^2^ on nickel foam.

### 2.3. Material Characterization

The structural characteristics of the quaternary CNCZO composite were examined using a Proto-AXRD Benchtop Diffractometer, (Proto Scientific Pvt. Ltd., Hyderabad, India) employing CuKα radiation across a 2θ scan range of 10° to 80°. X-ray photoelectron spectroscopy (JPS 9030 JEOL, Akishima, Japan) was used to analyze the surface composition and electronic states. To study the surface morphology of the electrode active material, a Field Emission Scanning Electron Microscope (FE-SEM) from (MIRA3 LMH, TESCAN, Brno, Czech Republic) was employed. Elemental composition analysis was carried out using energy-dispersive X-ray spectroscopy (EDS), integrated with the FE-SEM system, and facilitated by an Oxford Instruments EDS detector. Morphological insights were further verified using transmission electron microscopy (Talos F200i, Thermo Fisher Scientific, Seoul, Republic of Korea).

### 2.4. Measurements of Electrochemical Performance

Electrochemical characterization was performed using a standard three-electrode setup to evaluate the supercapacitive performance of the synthesized electrode material. All measurements were conducted on a CHI 660C electrochemical workstation, Austin, Texax, USA. The analysis involved various techniques, including cyclic voltammetry (CV), galvanostatic charge–discharge (GCD), and electrochemical impedance spectroscopy (EIS). In the three-electrode configuration, the metal oxide-based material on different substrates served as the working electrode, while graphite and Ag/AgCl were used as the counter and reference electrodes, respectively. A 1 M KOH aqueous solution functioned as the electrolyte.

The specific capacitance of the electrode was determined from the GCD profiles using the following expression:

(1) $$Cs=\frac{I\times \Delta t}{\Delta m\times \Delta V}$$

where I represents the current density as the discharge time (s), Δm is the mass of active materials (g), and ΔV is the potential window. The power density and energy density are calculated from the galvanic charging–discharging curves based on the equation as follows:

(2) $$E_{g}=\frac{1}{2}\frac{Cs\times \Delta V^{2}}{3600}$$

(3) $$P_{g}=\frac{E}{\Delta t}\times 3600$$

where E_g_ is energy density and P_g_ power density are in terms of Wh/kg and W/kg respectively.

## 3. Results and Discussion

**Figure 1:**
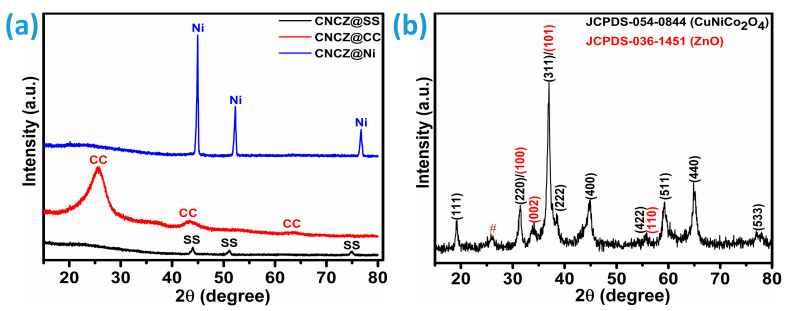
XRD analysis, (**a**) diffraction patterns of CNCZO@SS, CNCZO@CC, and CNCZO@Ni, and (**b**) diffraction patterns of the powder scratched from the CNCZO@Ni-foam sample.

To evaluate the phase composition and crystalline quality of the synthesized electrode material, X-ray diffraction (XRD) measurements were performed. Figure 1a presents the XRD patterns of the quaternary CNCZO nanostructures grown hydrothermally on different conductive substrates CC, SS mesh, and Ni-foam. The CNCZO@Ni foam exhibited prominent reflections at approximately 44.89°, 52.15°, and 76.73°, corresponding to the (111), (200), and (220) planes of metallic nickel, as referenced by PDF #04-0850 [29]. Similarly, the CNCZO@SS mesh showed characteristic diffraction peaks near 43.98°, 51.14°, and 74.95°, attributed to the (111), (200), and (220) planes, in alignment with PDF #33-0397 [29]. For the CNCZO@CC, peaks detected at 25.58° and 43.30° are indicative of the (002) and (101) lattice planes, respectively, consistent with the XRD pattern of pristine carbon cloth [30]. Owing to the minimal loading of the active material, its XRD signals are relatively weak and completely masked by the dominant substrate peaks. Furthermore, overlapping peaks hinder clear identification of the individual crystalline phases of the deposited layer on the corresponding substrates. To validate the crystalline characteristics of the hydrothermally synthesized CoNiCuZn oxide, X-ray diffraction (XRD) was conducted on the scratched powder samples, as Figure 1b illustrates. The diffraction pattern revealed well-defined peaks, indicative of the formation of a crystalline quaternary oxide phase. Notable reflections appeared at 2θ angles near 19.45°, 31.25°, 37.16°, 45.02°, 55.85°, 59.58°, and 65.08°, which can be assigned to the (111), (220), (101), (311), (102), (400), (422), (200), (103), (511), and combined (200)/(400) planes. These assignments align with standard crystallographic data referenced from JCPDS file No. #54-0844 and #36-1451, affirm the successful synthesis of a mixed-metal quaternary oxide incorporating cobalt, nickel, copper, and zinc components. One unidentified small peak was noted at 2θ = 26°, mentioned by symbol #.

**Figure 2:**
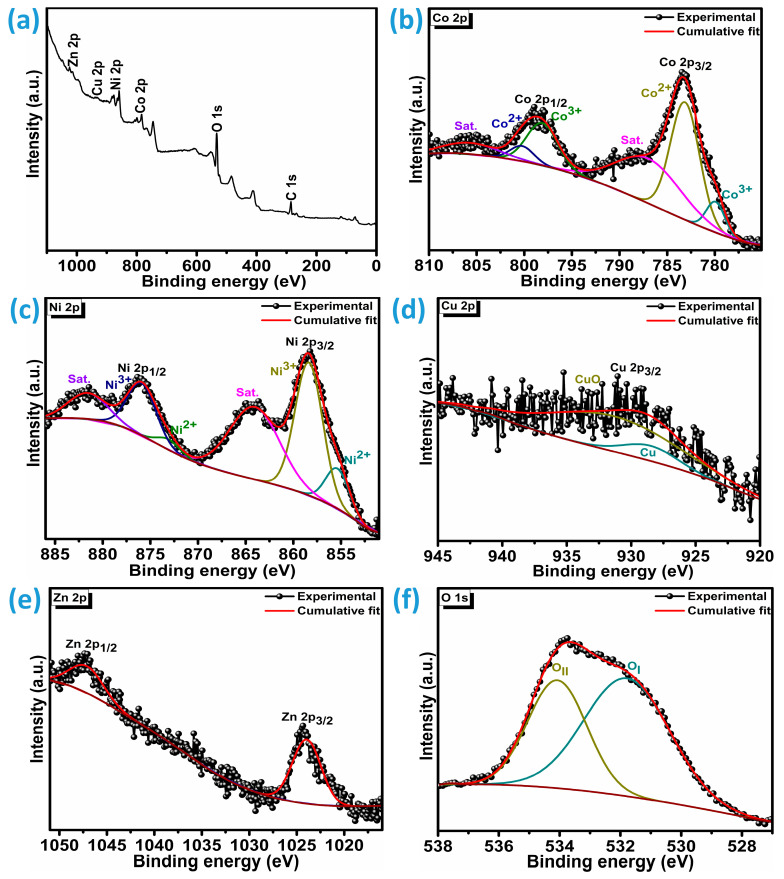
XPS analysis of CNCZO@Ni, (**a**) survey spectrum, (**b**) Co 2p, (**c**) Ni 2p, (**d**) Cu 2p, (**e**) Zn 2p, and (**f**) O 1s.

The chemical composition and surface electronic characteristics of CNCZO@Ni after calcination at 250 °C for 2 h were investigated using X-ray photoelectron spectroscopy (XPS), as depicted in Figure 2a. To further interpret the oxidation states of the constituent elements, Gaussian peak fitting was applied. The XPS spectrum in Figure 2b reveals two distinct peaks for cobalt, attributed to Co 2p_3/2_ and Co 2p_1/2_, located at binding energies of 783.30 eV and 798.92 eV, respectively. The Co 2p_3/2_ peak was further resolved into Co^2+^ 2p_3/2_ and Co^3+^ 2p_3/2_ components at 779.9 eV and 783.1 eV, respectively. Similarly, the Co 2p_1/2_ signal was deconvoluted into Co^2+^ and Co^3+^ components at 798.2 eV and 800.2 eV. The binding energy separation of approximately 15.6 eV between Co 2p_3/2_ and Co 2p_1/2_ is consistent with the coexistence of both Co^2+^ and Co^3+^ oxidation states. The satellite peaks are broad for both core levels, which are centered at 787.3 eV for 2p3/2 and 805.8 eV for 2p1/2, respectively [24,26,31]. Figure 2c presents the Ni XPS spectrum, showing two primary peaks at 858.5 eV and 876.1 eV, corresponding to the Ni 2p_3/2_ and Ni 2p_1/2_ core levels and shakeup satellites at 864.1 eV and 881.6 eV illustrates the presence of Ni^2+^ and Ni^3+^ species. Peak deconvolution of the Ni core levels reveals the presence of two distinct oxidation states: Ni^2+^ and Ni^3+^. For the Ni 2p_3/2_ level, the Ni^2+^ component appears at a binding energy of 855.5 eV, while the corresponding Ni 2p_1/2_ peak is observed at 872.9 eV. In contrast, the Ni^3+^ state is identified at 858.4 eV and 876.1 eV within the 2p_3/2_ and 2p_1/2_ levels, respectively [32,33]. As illustrated in Figure 2d, the Cu 2p_3/2_ peak is located at 929.7 eV, deconvolution implies the core level 2p_3/2_ includes two peaks corresponding to Cu (928.5 eV), and CuO (931.4 eV). The presence of CuO, as evidenced by peaks at 931.4 eV, suggests that surface copper is readily susceptible to oxidation under ambient conditions [34]. In Figure 2e, the Zn XPS profile displays the Zn 2p_3/2_ and Zn 2p_1/2_ peaks at binding energies of 1024.51 eV and 1047.33 eV, respectively, which are consistent with standard Zn^2+^ signals [35,36]. Figure 2f shows the O 1s XPS spectrum, which comprises two peaks: O_I_ and O_II_. O_I_ is centered at 531.8 eV, and this peak is typically attributed to oxygen involved in metal-oxygen-metal (M–O–M) bonding environments or lattice oxygen species. The O_II_ (534.1 eV) peak reflects the chemisorbed oxygen or defect sites in the quaternary metal oxide [37]. From these observations, it can be concluded that the CNCZO material is a mixed quaternary system incorporating cobalt, nickel, copper, and zinc species, all coordinated with oxygen.

**Figure 3:**
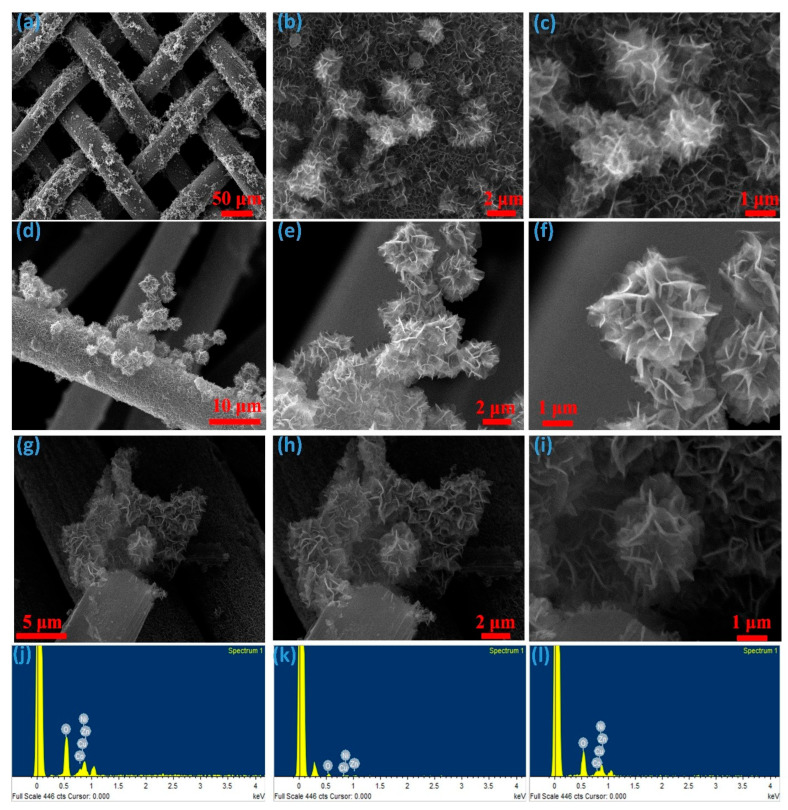
Morphological (FE-SEM) and elemental (EDS) analysis, (**a**–**c**) CNCZO@SS, (**d**–**f**) CNCZO@CC, (**g**–**i**) CNCZO@Ni, and (**j**–**l**) EDS spectrum of CNCZO@SS, CNCZO@CC, and CNCZO@Ni respectively.

The morphological characteristics of the electrode material play a pivotal role in defining the electrochemical performance of supercapacitors. Following the synthesis of CNCZO on various substrates, a three-dimensional structure resembling a dual-petal hibiscus flower emerges, markedly enhancing the available surface area. This kind of architecture of active material improves ion diffusion and electron transfer kinetics. As Figure 3a–c illustrates, CNCZO’s flower-like architecture is made up of nanopetals that are grown on stainless steel (SS) mesh. The porous nature of the SS mesh offers a large area conducive to nucleation. Figure 3d–f shows CNCZO nanostructures integrated onto carbon cloth. The fabric’s mesh-like, porous configuration supports effective material deposition and promotes three-dimensional growth, facilitating charge transport. In Figure 3g–i, CNCZO nanoflowers are deposited onto nickel foam. The foam’s interconnected, sponge-like structure with abundant pores and cross-linking provides ample surface for nucleation and supports uniform nanoflower formation. The high surface-to-volume ratio of the nickel foam reduces interfacial resistance between the active material and electrolyte, thereby enhancing capacitive behavior. FE-SEM images reveal that the CNCZO nanostructures form a three-dimensional flower-like architecture, composed of integrated petals that are uniformly distributed across the electrode surfaces. These nanopetals are interconnected and fused, forming continuous conductive pathways that allow efficient penetration of electrolyte ions. Energy Dispersive X-ray Analysis (EDAX) was employed to determine the elemental composition of the synthesized material. As seen in Figure 3j–l, the EDAX spectra and corresponding elemental maps confirm the presence of cobalt, nickel, copper, zinc, and oxygen. Notably, no signals from extraneous elements were observed, validating the successful and uncontaminated synthesis of CNCZO nanostructures in the desired stoichiometry.

**Figure 4:**
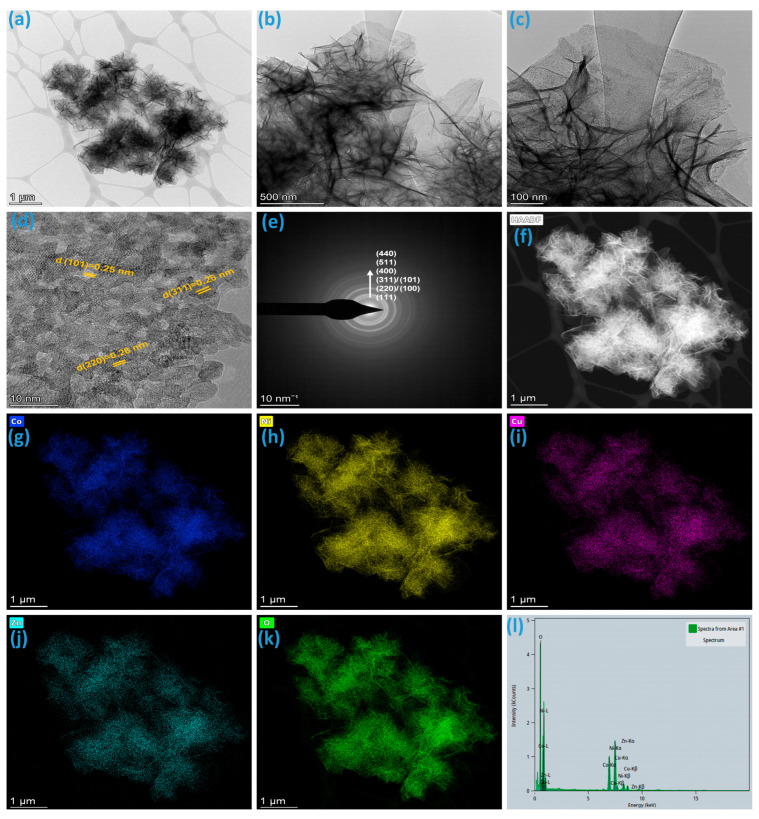
TEM analysis of CNCZO@Ni sample, (**a**–**c**) TEM images at different magnifications, (**d**) HRTEM image, (**e**) SAED pattern, (**f**–**k**) HAADF and elemental mapping of Co, Ni, Cu, Zn respectively, and (**l**) EDS spectrum.

In Figure 4a–c, TEM images offer a closer examination of the CNCZO nanoflowers deposited on nickel foam, revealing intricate internal morphology. The images display that the nanoflowers are composed of thin, sheet-like petals exhibiting significant bending, curling, and crumpling. These morphological features arise from the inherently larger lateral dimensions of the nanosheets relative to their thickness, leading to structural distortion during growth or assembly. This wrinkled configuration contributes to an enlarged surface area and generates more exposed active sites, which is beneficial for electrochemical interactions. This observation confirms the flower-like assembly observed in FE-SEM. These wrinkles increase the effective surface area and can facilitate enhanced electrolyte penetration and ion diffusion. The high-resolution TEM (HRTEM) image (Figure 4d reveals that each petal of the nanoflowers consists of ultrathin, and crystalline nanoparticles. The lattice fringes observed in the HRTEM images indicate the high crystallinity of the CNCZO phase, with well-defined interplanar spacings of 0.25 nm and 0.28 nm corresponding to specific (311), (101), and (220) crystal planes, further validating the formation of a crystalline material observed in XRD. Selected Area Electron Diffraction (SAED) patterns (Figure 4e) display distinct diffraction rings, suggesting a polycrystalline nature with randomly oriented nanocrystals. The uniform contrast across the petals indicates homogenous elemental distribution, while the well-resolved edges highlight the sharp and clean morphology of the nanopetals. Elemental mapping (Figure 4f–k) and EDS (Figure 4l) from TEM further confirm the wrinkled nanopetals are composed of Co, Ni, Cu, Zn, and O elements. These features support the conclusion that the CNCZO nanoflowers are composed of interconnected nanopetals, contributing to a high surface area and facilitating efficient electron and ion transport during electrochemical processes.

**Figure 5:**
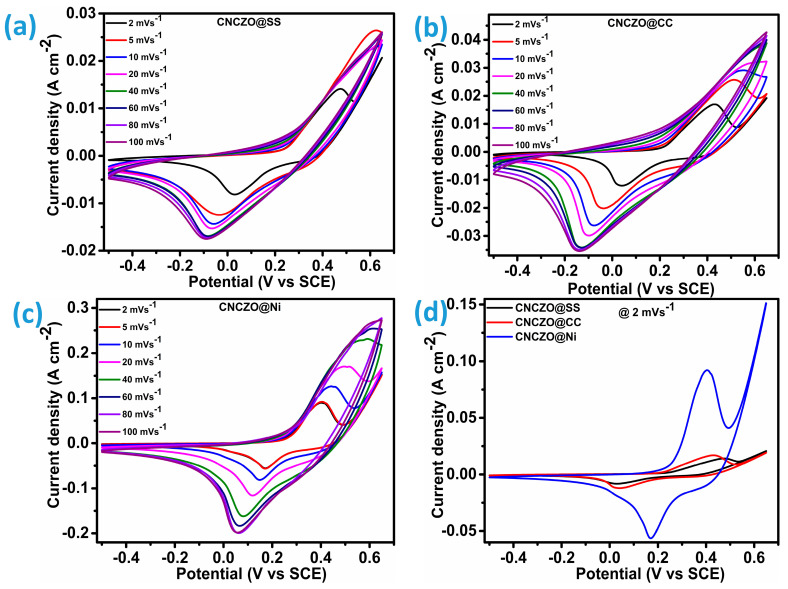
Cyclic voltammetry at different scan rates (**a**) CNCZO@SS, (**b**) CNCZO@CC, (**c**) CNCZO@Ni, and (**d**) comparative CV at 2 mV/s for all electrodes.

The electrochemical behavior of quaternary CNCZO nanostructures, synthesized as petal-like formations on different conductive substrates CC, SS mesh, and Ni foam, was investigated. These substrates were directly employed as working electrodes in a three-electrode setup using a 1 M KOH aqueous electrolyte. The evaluation involved cyclic voltammetry (CV), galvanostatic charge–discharge (GCD), and impedance spectroscopy (EIS) measurements. The study capitalizes on both the morphological characteristics and the multi-component composition of the CNCZO electrodes, which collectively enhance their electrochemical response. CV testing was specifically performed to examine the redox interactions between the metal ions and the electrolyte. The cyclic voltammograms of the developed electrode samples, as presented in Figure 5a–c, were recorded over a potential window ranging from −0.5 V to 0.65 V at varying scan rates. The prepared electrode exhibits distinct redox peaks in its cyclic voltammetry (CV) curves, which are attributed to the likely quasi-reversible redox interactions between the CNCZO material and hydroxide (OH^−^) ions present in the KOH electrolyte [38,39,40,41,42,43]. The possible reversible reaction mechanism can be illustrated as [40]:

(4) $$\left(CuZn\right)NiCoO+H_{2}O+{OH}^{-}⟷CoOOH+NiOOH+2e^{-}$$

(5) $$CoOOH+{OH}^{-}⟷{CoO}_{2}+H_{2}O+e^{-}$$

Despite the increased scan rate, the cyclic voltammetry (CV) profiles maintain their characteristic form, reflecting the electrode’s strong electrochemical reversibility and impressive rate performance. A proportional rise in peak current density with increasing scan rates highlights the sustained efficiency of electron transport, even under accelerated conditions [44,45]. The unique nanopetal-like structure plays a crucial role in promoting electrolyte ion diffusion through the intricate petal network. This architecture offers an expanded surface area and enhanced connectivity, both of which support superior ion mobility and contribute to the system’s capacitive response. The well-defined redox peaks observed in the CV curves confirm the presence of pseudocapacitive behavior, a key feature of Faradaic processes. As Figure 5d illustrates, among the evaluated substrates, CNCZO@Ni foam demonstrates a significantly larger CV area compared to CNCZO@SS mesh and CNCZO@CC, indicating more efficient charge propagation and resulting in higher specific capacitance.

By systematically examining cyclic voltammetry (CV) data across varying scan rates, it becomes possible to differentiate between capacitive and faradaic behaviors. In faradaic reactions, the peak current demonstrates a proportional relationship to the square root of the scan rate, reflecting diffusion-limited charge transfer processes. Conversely, for capacitive mechanisms, such as electric double-layer formation or surface redox, the current increases linearly with the scan rate, indicating surface-controlled kinetics. This distinction is vital for tailoring electrochemical systems to desired performance characteristics. Typically, the current response follows a power-law dependency on the scan rate, often described using Dunn’s power law, as expressed by the equation [46]:

(6) $$i=kv^{b}$$

where i is current, v is the scan rate, and the exponent b reveals the dominant charge storage mechanism.

**Figure 6:**
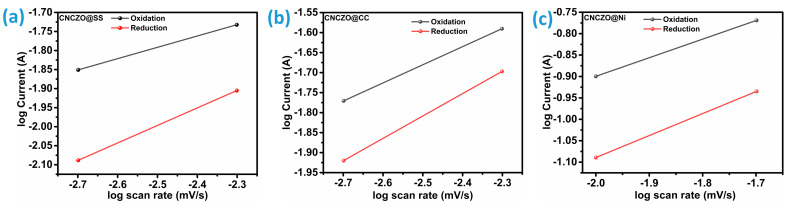
The plot of log (peak current (A)) vs. log (scan rate (mV/s)), (**a**) CNCZO@SS, (**b**) CNCZO@CC, and (**c**) CNCZO@Ni.

Estimation of the b value is possible by plotting the (log (i)) peak current density vs scan rate; Figure 6a–c illustrates this for CNCZO@SS mesh, CNCZO@CC, and CNCZO@Ni foam, respectively. This b value serves as a key indicator of the predominant charge storage mechanism, whether it is primarily capacitive or faradaic (involving electron transfer). Moreover, it provides valuable information about the kinetics of charge storage at different potentials and scan rates. A b value ranging from 0.5 to 1 reflects distinct electrochemical behaviors: A b value of 0.5 typically points to diffusion-limited reactions, similar to those found in battery-type processes. A value near 1 suggests surface or near-surface charge-transfer mechanisms indicative of faradaic processes governed by capacitive kinetics [47]. For anodic diffusion at scan rates below 40 mV/s, the calculated b values are 0.4521 for CNCZO@SS Mesh, 0.45316 for CNCZO@CC, and 0.43381 for CNCZO@Ni foam. These values, all close to 0.5, imply that diffusion plays a significant role in the charge storage process. From these findings, it can be inferred that all three electrode materials predominantly follow a diffusion-controlled charge storage pathway, a hallmark of pseudocapacitive behavior.

**Figure 7:**
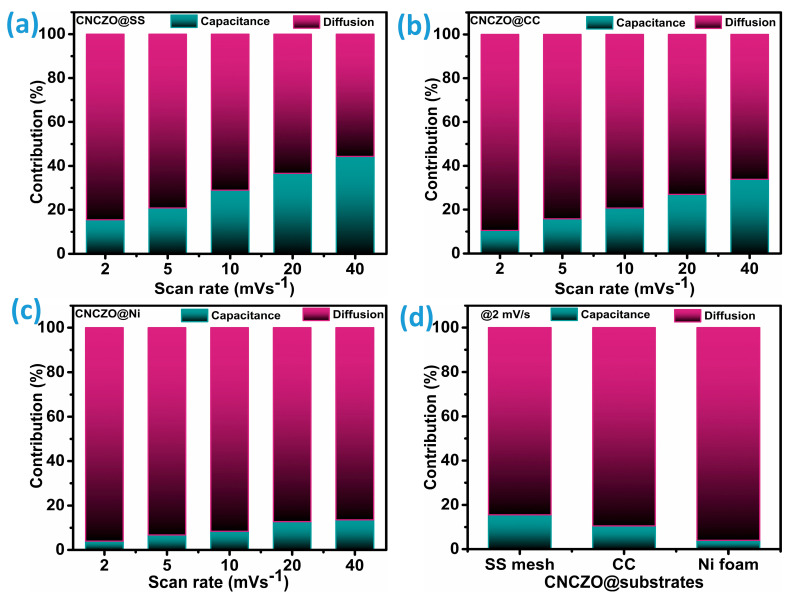
Estimated capacitance and diffusion-controlled charge storage contribution for CNCZO on different substrates (**a**) CNCZO@SS mesh, (**b**) CNCZO@CC, (**c**) CNCZO@Ni foam, and (**d**) combined plot at 2 mV/s scan rate.

To gain deeper insight into the individual contributions of diffusion-controlled and capacitive processes within the electrode, an extended form of Dunn’s power law is employed as follows [46,47]:

(7) $$i\left(v\right)=k_{1}v+k_{2}v^{0.5}$$

where i represents the current under fixed potential (V), and k_1_ and k_2_ are constants. By plotting the relationship between current (i) and the square root of the scan rate (ν^0.5^), the slope (k_1_) and the y-intercept (k_2_) can be extracted. This method enables a clear depiction of the prevailing charge storage mechanisms within the total electrochemical response, as Figure 7a–c demonstrates. Figure 7d presents a comparison of the charge storage contribution ratios for CNCZO@SS mesh, CNCZO@CC, and CNCZO@Ni foam at a scan rate of 2 mV/s. As evident from Figure 7a–c, CNCZO on all three substrates exhibits a largely diffusion-driven behavior at lower scan rates. However, as the scan rate increases, the capacitive portion becomes more prominent, indicating a progressive enhancement in capacitive involvement in the overall storage process. This trend is linked to the increased mobility of electrolyte ions at higher scan rates, which facilitates faster surface redox interactions and restricts ion penetration into the electrode matrix. For the CNCZO@SS electrode, the diffusion-controlled share drops from 85% at 2 mV/s to 56% at 40 mV/s. Similarly, the CNCZO@CC electrode shows a decline in this contribution from 89% to 66% over the same scan rate range. In contrast, CNCZO@Ni exhibits a much smaller reduction in diffusion-dominated behavior, with values decreasing only from 96% to 86.4%, suggesting that pseudocapacitive processes remain dominant over a wide range of scan rates [48].

**Figure 8:**
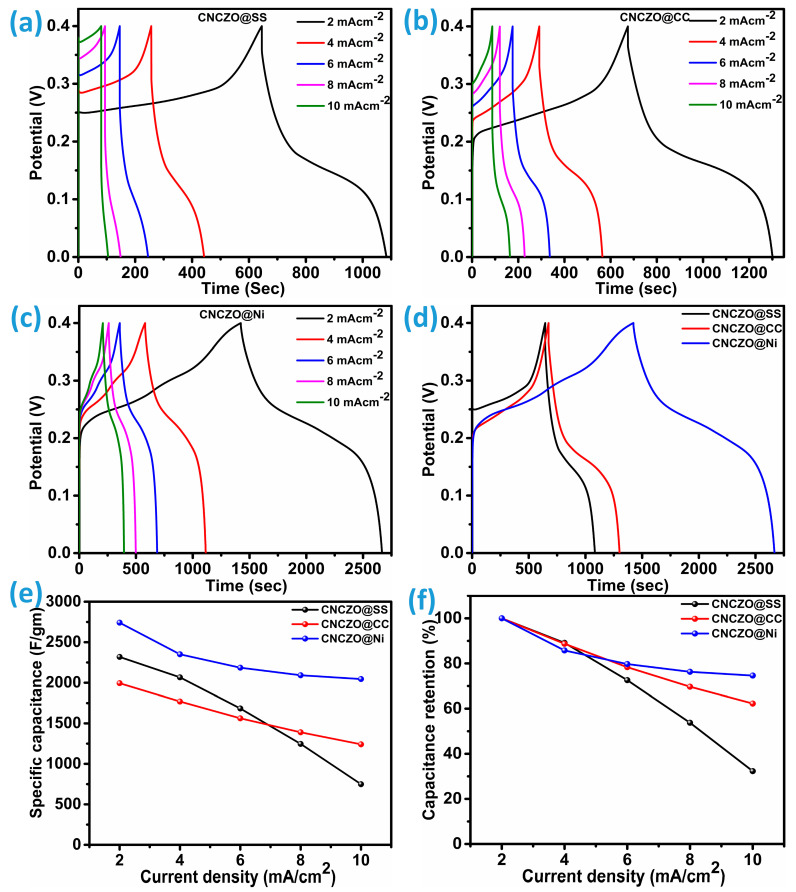
GCD curve at different current densities (**a**) CNCZO@SS, (**b**) CNCZO@CC, (**c**) CNCZO@Ni, and (**d**) comparative GCD for CNCZO deposited on CC, SS mesh, and Ni-foam at the current density of 2 mA/cm^2^, (**e**) specific capacitance vs current density for all electrodes, and (**f**) capacitance retention vs current density for all electrodes.

Galvanostatic charge–discharge (GCD) analysis serves as a reliable technique to evaluate the electrochemical behavior of pseudo-supercapacitors. The gravimetric performance of the fabricated porous electrodes was investigated through their charge/discharge characteristics, as illustrated in Figure 8a–c. GCD measurements were performed on all three electrode samples across a range of current densities, 2, 4, 6, 8, and 10 mA/cm^2,^ within an operational voltage window of 0 to 0.4 V. The results indicate a clear trend: as the applied current density decreases, the discharge duration extends for each electrode, highlighting favorable conditions for supercapacitor functionality. The presence of non-linear charge/discharge profiles points to pseudocapacitive behavior, suggesting the involvement of quasi-reversible redox reactions occurring at the electrode-electrolyte boundary [39,40]. At higher current densities, a noticeable reduction in available redox-active sites leads to diminished specific capacitance and a pronounced voltage drop. Despite this, the hybrid quaternary (CNCZO) metal oxide exhibits excellent rate capability, indicating improved ion transport and electrolyte penetration, which collectively enhance its overall capacitance performance. Moreover, a steeper charging slope at 10 mA/cm^2^ reflects a decline in energy storage capacity, attributed to constrained ion diffusion and suboptimal utilization of active material.

In contrast with CNCZO@SS and CNCZO@CC, the Ni foam-based CNCZO electrode displays a more gradual charge–discharge profile, with even current density going towards higher values, indicative of higher charge retention and superior capacitance, particularly under lower current conditions. During discharge, the rate of voltage decline serves as a direct indicator of capacitance slower decay corresponds to greater energy storage capability and vice versa. We compare the GCD curves of CC, SS mesh, and Ni-foam at 2 mA/cm^2^ current density (Figure 8d). The CNCZO@Ni-foam indicates the highest discharge time among the other two, indicating excellent energy storage capacity and lower resistance. The specific capacitance calculated from the formula, which is mentioned in Equation (1), at 2 mA/cm^2^ is 2318.5 F/g, 1993.7 F/g, and 2741.3 F/g for CNCZO@SS mesh, CNCZO@CC, and CNCZO@Ni foam, respectively. Additionally, the specific capacitance values corresponding to various current densities were determined for each CNCZO-based electrode and are presented in Figure 8e. A comparative analysis of capacitance retention, as shown in Figure 8f, was conducted across the current density range of 2 mA/cm^2^ to 10 mA/cm^2^. Among the tested samples, the CNCZO-Ni electrode exhibited the most stable performance, maintaining approximately 74.6% of its initial capacitance. In contrast, the CNCZO@CC electrode preserved 62.2% of its capacitance under the same conditions, while the CNCZO@SS electrode demonstrated a substantial decline, retaining only 32.3%. This trend underscores the superior rate capability of the CNCZO-Ni configuration, which may be attributed to enhanced electronic conductivity and stronger interfacial contact between the active material and the Ni substrate. The moderate retention observed in CNCZO@CC suggests partial efficacy in maintaining electrochemical performance under increased current stress, potentially due to better flexibility and surface area characteristics of carbon cloth. Conversely, the poor performance of CNCZO@SS indicates significant limitations, possibly arising from lower conductivity and weaker interaction with the active material, which collectively hinder efficient charge transfer at higher current densities.

**Figure 9:**
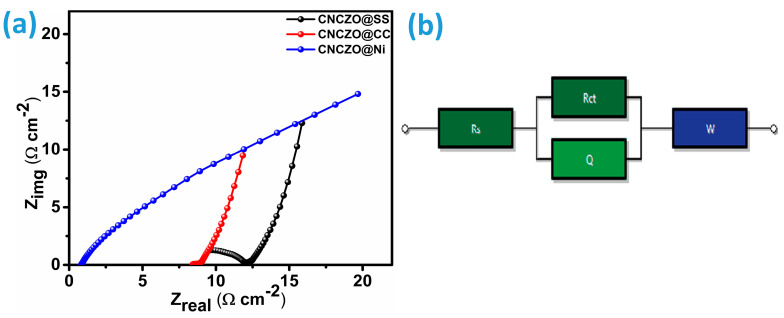
(**a**) EIS for CNCZO@SS, CNCZO@CC, and CNCZO@Ni and (**b**) equivalent circuit.

Electrochemical Impedance Spectroscopy (EIS) is an essential diagnostic tool used to investigate electrical conductivity, ionic movement, charge transfer dynamics, and the origins of capacitive characteristics in supercapacitor electrodes [49]. In this study, EIS measurements were performed across a wide frequency spectrum, ranging from 0.01 Hz to 1 MHz. The data were visualized using Nyquist plots (Figure 9), which present the real component of impedance (Z′) along the x-axis and the imaginary component (Z″) along the y-axis [49,50]. A typical Nyquist plot reveals two primary regions: a semicircular arc at high frequencies and an inclined linear segment at low frequencies. The high-frequency semicircle reflects the combined effects of electrolyte resistance and charge transfer resistance at the electrode–electrolyte interface, while the linear portion at lower frequencies is associated with ion diffusion and double-layer capacitance. Additionally, the occurrence of a third region is possible; a linear region inclined at approximately 45° indicates the presence of Warburg impedance, which is characteristic of diffusion-limited processes [47,51,52]. Using equivalent circuit modeling (Figure 9b), the resistance parameters were extracted for each CNCZO electrode. The values of Rs, Rct, and Warburg’s resistance (W) for CNCZO@SS mesh as 6.971 Ω/cm^2^, 9.288 Ω/cm^2^, 0.3763 Ω/cm^2^, for CNCZO@CC as 0.001 Ω/cm^2^, 32.23 Ω/cm^2^, 1.056 Ω/cm^2^, and for CNCZO@Ni foam as 0.7416 Ω/cm^2^, 4.561 Ω/cm^2^, 0.05316 Ω/cm^2^. Among the three electrodes studied, the CNCZO@Ni foam electrode demonstrated the lowest charge transfer resistance, indicating enhanced ion mobility and reduced resistive losses, which contributed to superior electrochemical performance.

**Figure 10:**
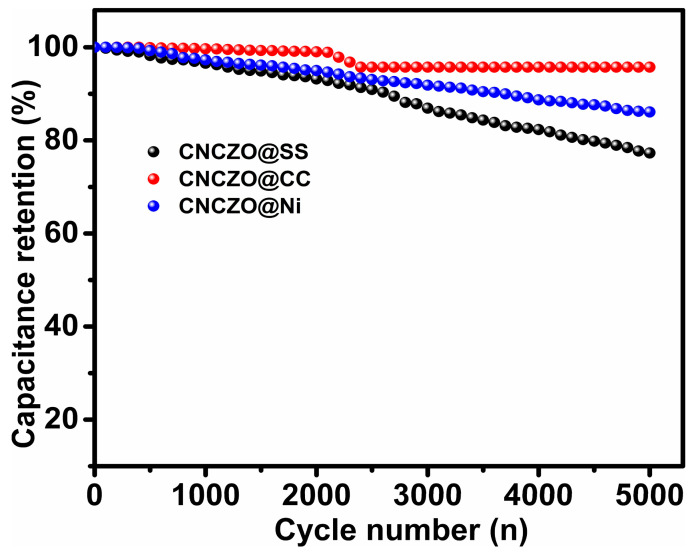
Stability test for CNCZO@SS, CNCZO@CC, and CNCZO@Ni.

The long-term cycling performance of the CNCZO electrodes was assessed over 5000 charge–discharge cycles (Figure 10). The capacitance retention after cycling was recorded as 77.3% for the CNCZO@SS mesh, 95.6% for the CNCZO@CC, and 86.1% for the CNCZO@Ni foam electrode. Although the Ni foam electrode initially demonstrated lower resistance and outperformed others in early-stage electrochemical behavior, it succeeded in retaining the median capacitance among the group after prolonged cycling, indicating strong durability. The SS mesh maintained moderate stability with a retention rate of 77.3%, whereas the carbon cloth exhibited the most consistent performance, preserving 95.6% of its initial capacitance, and suggesting excellent endurance. These results collectively indicate that the CNCZO@Ni foam electrode combines robust initial performance with reliable long-term stability, making it a promising candidate for high-efficiency supercapacitor applications.

**Figure 11:**
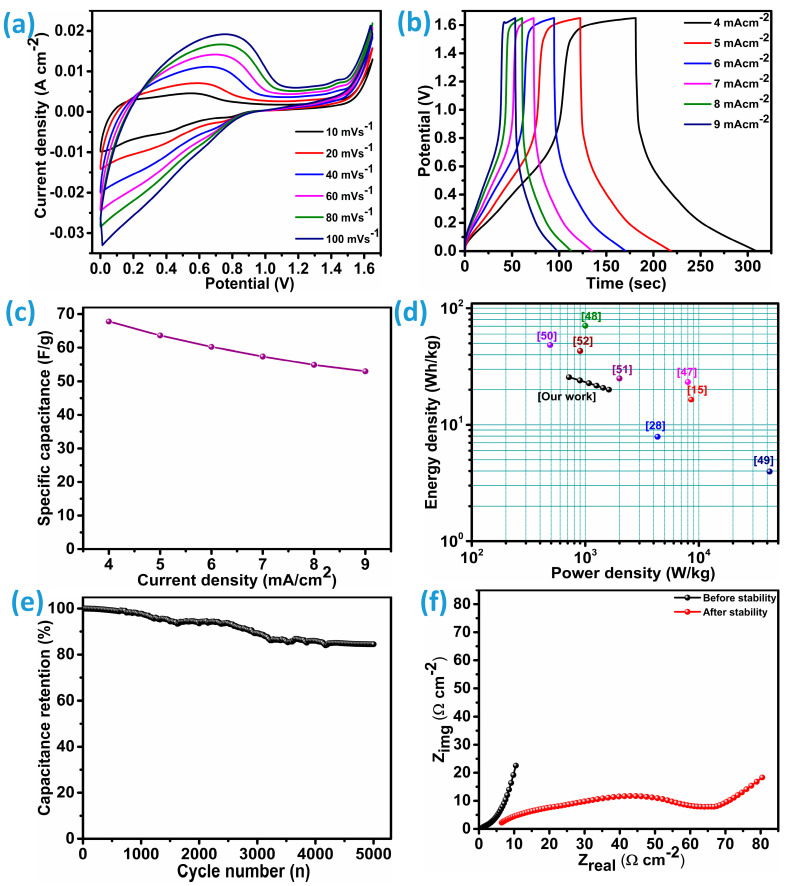
Symmetric device (CNCZO@Ni) electrochemical assessment, (**a**) the CV curves at various scan rates, (**b**) GCD curves at varying current densities, (**c**) specific capacitance vs scan rate, (**d**) Ragone plot, (**e**) cycle stability for the device, and (**f**) EIS for the device before and after stability.

CNCZO-coated nickel foam (CNCZO@Ni foam) electrodes were employed as both the anode and cathode in a symmetric supercapacitor device. The system utilized a 1 M KOH aqueous solution as the electrolyte, with a flexible polyvinyl alcohol (PVA) membrane serving as the separator. The electrochemical performance of the device was systematically analyzed using techniques such as cyclic voltammetry (CV), galvanostatic charge–discharge (GCD), electrochemical impedance spectroscopy (EIS), and long-term cycling tests. As Figure 11a illustrates, CV curves recorded at multiple scan rates demonstrated consistent profiles, highlighting efficient ion transport at the electrode–electrolyte interface. The minimal distortion of the CV curves across varying scan rates indicates high electrochemical reversibility and stable ion diffusion. Figure 11b illustrates the galvanostatic charge–discharge (GCD) curves measured across a range of current densities, from 4 to 9 mA/cm^2^. These profiles reveal the charging and discharging characteristics of the device. The nearly linear form of the curves suggests behavior typical of an ideal capacitor, highlighting minimal internal resistance and efficient charge transport during cycling. Moreover, the device achieves a notable specific capacitance of 67.7 F/g at 4 mA/cm^2^ and 53 F/g at the current density of 9 mA/cm^2^ (Figure 11c). At such a high current density, i.e., 4 mA/cm^2^, it delivers an energy density of 25.6 Wh/kg and an impressive power density of 717.4 W/kg; at 9 mA/cm^2^, it becomes 20 Wh/kg and 1614.1 W/kg. This implies that the symmetric device was able to deliver a good rate capability of 78.2% over the wide range of current density. These metrics collectively demonstrate the device’s potential for applications requiring both high energy and high power performance. The device exhibits superior performance relative to numerous symmetric counterparts cited in recent studies and achieves performance levels comparable to other similar devices. Comparative demonstration of the symmetric devices studied in literature with CNCZO@Ni//CNCZO@Ni was illustrated through the Ragone plot (Figure 11d). The CNCZO electrodes demonstrated an exceptionally high specific capacitance of 2741.3 F/g when tested in a three-electrode setup. However, upon integration into a symmetric two-electrode device, this value decreased sharply to 67.7 F/g. This reduction arises mainly from the differences between the idealized measurement conditions in the three-electrode system and the practical constraints of the full-cell device. In the three-electrode arrangement, the performance of the working electrode is evaluated independently against a stable reference electrode, with a counter electrode that does not limit current flow. This configuration minimizes internal resistances and enables nearly full utilization of the active material, often measured at low mass loadings, which enhances the apparent specific capacitance. Conversely, the symmetric device requires both electrodes to function simultaneously, which inherently increases the internal resistance due to the combined effects of two electrodes, the separator, and current collectors. To ensure proper cell balancing, equal mass loading on both electrodes is necessary, increasing the overall active material mass and thereby lowering the specific capacitance per unit weight. Moreover, the device’s compact architecture can restrict ion transport and limit electrolyte infiltration, further decreasing the effective utilization of the electrode material. These practical limitations collectively explain the significant drop in capacitance observed in the symmetric cell configuration.

Subsequently, the durability of the device under continuous operation was investigated using GCD over multiple cycles; the results are depicted in Figure 11e. The device showed remarkable electrochemical stability, retaining approximately 84.5% of its initial capacitance after 5000 cycles at a current density of 25 mA/cm^2^. This level of retention highlights the robustness of the CNCZO@NF electrode-based symmetric device and its potential for deployment in practical energy storage systems that demand longevity and reliability. To further evaluate the impedance features of the device, electrochemical impedance spectroscopy (EIS) was employed. The corresponding Nyquist plots recorded before and after the stability evaluation are presented in Figure 11f. These spectra provide insights into the ionic resistance (Rs) and the device’s overall electrical conductivity. The consistently low Rs values in both conditions (0.48 Ω/cm^2^-before stability and 6.4 Ω/cm^2^-after stability) indicate minimal interfacial charge transfer resistance, underscoring the system’s effectiveness in facilitating ion mobility and electrical conduction. These low resistance measurements also suggest that the device sustains a high conductivity level even after extended cycling, a favorable trait for long-term energy storage applications. Overall, this detailed analysis confirms that CNCZO@NF is a promising electrode material for symmetric supercapacitors, offering strong charge–discharge reversibility, excellent ionic and electronic conductivity, and superior long-term cycling performance.

## 4. Conclusions

The development of 3-D curled and scrunched petal hibiscus flowers of mixed quaternary CNCZO on various porous substrates presents a promising advancement for high-performance supercapacitors. The innovative structure and composition are expected to deliver superior energy storage capabilities, expanding the frontiers of current technology in energy storage devices. Further research and development are essential to overcome the challenges and fully realize the potential of these novel materials. We have successfully deposited CNCZO mixed quaternary oxide on various substrates, including SS mesh, carbon cloth, and Ni foam, using the facile hydrothermal method. From structural analysis, it was found that a mixed quaternary metal CNCZO was formed. The nano-petal structure of the deposited oxide, characterized by its expansive surface area and streamlined path for electrolyte diffusion, along with the compatibility of Co, Ni, Cu, and Zn, contributes to the enhanced performance of the prepared electrodes. Among the substrates tested, the Ni foam electrode exhibited remarkable results due to its high porosity and interconnected channel morphology.

## Data Availability

The original contributions presented in this study are included in the article. Further inquiries can be directed to the corresponding author.
